# Haplotypes that include the *integrin alpha 11 *gene are associated with tick burden in cattle

**DOI:** 10.1186/1471-2156-11-55

**Published:** 2010-06-21

**Authors:** Laercio R Porto Neto, Rowan J Bunch, Blair E Harrison, Kishore C Prayaga, William Barendse

**Affiliations:** 1 Cooperative Research Centre for Beef Genetic Technologies, University of New England, Armidale, NSW 2351, Australia; 2 CSIRO Livestock Industries, Queensland Bioscience Precinct, 306 Carmody Road, St. Lucia, QLD 4067, Australia; 3 The University of Queensland, School of Animal Studies, St. Lucia, QLD 4072, Australia

## Abstract

**Background:**

Infestations on cattle by the ectoparasite *Boophilus (Rhipicephalus) microplus *(cattle tick) impact negatively on animal production systems. Host resistance to tick infestation has a low to moderate heritability in the range 0.13 - 0.64 in Australia. Previous studies identified a QTL on bovine chromosome 10 (BTA10) linked to tick burden in cattle.

**Results:**

To confirm these associations, we collected genotypes of 17 SNP from BTA10, including three obtained by sequencing part of the *ITGA11 *(*Integrin alpha 11*) gene. Initially, we genotyped 1,055 dairy cattle for the 17 SNP, and then genotyped 557 Brahman and 216 Tropical Composite beef cattle for 11 of the 17 SNP. In total, 7 of the SNP were significantly (*P *< 0.05) associated with tick burden tested in any of the samples. One SNP, ss161109814, was significantly (*P *< 0.05) associated with tick burden in both the taurine and the Brahman sample, but the favourable allele was different. Haplotypes for three and for 10 SNP were more significantly (*P *< 0.001) associated with tick burden than SNP analysed individually. Some of the common haplotypes with the largest sample sizes explained between 1.3% and 1.5% of the residual variance in tick burden.

**Conclusions:**

These analyses confirm the location of a QTL affecting tick burden on BTA10 and position it close to the *ITGA11 *gene. The presence of a significant association in such widely divergent animals suggests that further SNP discovery in this region to detect causal mutations would be warranted.

## Background

Tick infestation has a detrimental impact on animal production and ticks are one of the main vectors of pathogenic micro-organisms of veterinary and zoonotic importance [1,2]. deCastro [3] estimated global economic losses caused by tick and tick-borne diseases to the cattle industry in the range of US$18 billion per year.

Tick burdens are influenced by the genetic constitution of the host. Heritability (*h^2^*) estimates of tick burdens due to the ixodid tick *Boophilus (Rhipicephalus) microplus *in Australia range from *h^2 ^*= 0.13 - 0.64 [4-7] depending upon the season and breed of cattle analysed. In the animals used in this study, the heritability was *h^2 ^*= 0.37 (s.e. = 0.02) in the taurine animals and *h^2 ^*= 0.15 (s.e. = 0.10) in the Brahman animals [8,9]. Generally animals of zebu ancestry such as the Brahman carry an order of magnitude fewer ticks than animals of pure taurine origin such as the Hereford or Charolais [10-12]. Twenty one days after artificial infestations of 20,000 tick larvae, Brahman breed cattle will carry around 100 engorged ticks while taurine cattle will carry between 1-2 thousand engorged ticks [10].

Previous genetic studies found that the bovine leucocyte antigens (BoLA) were associated with tick burden [13,14] and these associations have more recently been confirmed using DNA polymorphisms [15-17], but the same allele has not always been associated with reduced tick numbers limiting the use of those markers in different populations. Whole genome scans using DNA microsatellites in linkage analyses have identified a small number of QTL associations [18,19] and a low density genome wide association study (GWAS) identified single nucleotide polymorphisms (SNP) associated with tick burden in several regions of the genome [20]. So far, no DNA marker or haplotype has shown a consistent effect across different breeds for the number of ticks that animals carry. Bovine chromosome 10 (BTA10) was found linked to tick burden in both a microsatellite whole genome scan and a low density GWAS [18,20]. In that GWAS, three SNP (rs29025985, rs29025981 and rs29025982) at approximately 15 Mb in the Btau 4.0 assembly [21] were associated with tick counts.

To determine whether the region on BTA10 (~15 Mb) showed significant associations to tick burden, two cattle samples 1) taurine dairy cattle of the dairy tick experiment (DTE) and 2) zebu and zebu-derived beef cattle from northern Australia in the tick zone, consisting of Brahman (BRM) and Tropical Composite (COM) cattle were used. We genotyped 17 SNP in the DTE sample and 11 of them in the BRM and COM samples, including 2 SNP from the GWAS [20] and SNP we identified from sequencing part of the *ITGA11 *(*Integrin alpha 11*) gene. Our aims were to replicate the association of the BTA 10 region to tick burden in independent samples, determine whether it was found in different types of cattle, estimate the size of the genetic effect in a large sample, and narrow down the region associated with tick burden.

## Results

We collected SNP from a variety of databases and sequenced part of the *ITGA11 *gene to identify more SNP. We identified 26 SNP in *ITGA11 *by sequencing exons 6-9 and adjacent introns in 16 animals from 4 different breeds. There were no differences in the coding sequence between taurine animals but there was one synonymous mutation segregating in BRM animals (Additional file 1). We chose 3 of the 26 SNP for further analysis based on their distribution across the sequenced region and minor allele frequency in all breeds of the panel sequenced. Some of the SNP from public databases were not polymorphic in any individuals in our sample. Of the 16 SNP used from the genome assembly database ftp://ftp.hgsc.bcm.tmc.edu/pub/data/Btaurus/, 15 were monomorphic when genotyped in the DTE, BRM and COM animals and may be sequencing artefacts [22]. This resulted in 17 SNP that were genotyped using the DTE animals and 11 SNP genotyped using the BRM and COM animals (Table 1). Of these SNP, rs29025980 showed a highly significant (*P *< 0.0001) departure from Hardy Weinberg Equilibrium (HWE) in almost all groups tested (5 out of 8 breed types). Apart from rs29025980, deviations from HWE occurred at a low rate (14 out of 124 comparisons) and no more than 2 breed types were significantly out of HWE.

**Table 1:** Description of markers tested in dairy and beef cattle samples

Locus	Position Btau4.0	Ref^1^	Sample^2^	N^3^	p_0_^4^	HWE^5^ p-value
rs29027392	9844454	BTA	DTE	820	0.03	0.0080 (1 breed)

rs41613225	9874407	BTA	DTE	820	0.34	0.0103 (1 breed)

rs41664397	11938389	BTA	DTE	820	0.15	0.0053 (1 breed)

Ars-BFGL-NGS-70946	14471603	Illumina	DTE	819	0.38	0.0173 (1 breed)

rs29025980	14925193	BTA	DTE	875	0.23	< 0.05 (3 breeds)
			
			BRM	525	0.05	1.34e-38
			
			COM	196	0.27	2.36e-24

rs43616884	14937129	BTB	DTE	915	0.28	ns
			
			BRM	546	0.05	0.0277
			
			COM	207	0.20	ns

rs29025985	14943961	BTA (GWAS)	DTE	812	0.68	ns
			
			BRM	464	0.09	ns
			
			COM	186	0.37	ns

rs29025981	14944238	BTA (GWAS)	DTE	859	0.32	ns
			
			BRM	517	0.91	ns
			
			COM	201	0.63	ns

rs41594962	14979585	BTA	DTE	1042	0.29	0.0256 (1 breed)
			
			BRM	545	0.01	ns
			
			COM	207	0.20	ns

ss161109814	14996440	This paper	DTE	1037	0.40	ns
			
			BRM	526	0.06	ns
			
			COM	205	0.24	ns

ss161109807	15002496	This paper	DTE	1027	0.28	ns
			
			BRM	525	0.62	ns
			
			COM	210	0.49	ns

ss161109797	15003391	This paper	DTE	1032	0.04	0.0337 (1 breed)
			
			BRM	526	0.05	ns
			
			COM	212	0.03	ns

rs29023635	15034937	BTA	DTE	914	0.69	ns
			
			BRM	549	0.06	ns
			
			COM	213	0.41	ns

rs29023639	15035100	BTA	DTE	296	< 0.01	ns
			
			BRM	548	< 0.01	ns
			
			COM	181	0.01	ns

rs29014770	15102776	BTA	DTE	901	0.68	ns
			
			BRM	544	0.99	ns
			
			COM	202	0.72	ns

rs41657550	17147707	BTA	DTE	820	0.68	ns

Ars-BFGL-NGS-25507	18216232	Illumina	DTE	819	0.75	< 0.05 (2 breeds)

^1 ^Database in which the markers were described, BTA - BCM Interbreed SNP, BTB - BCM Genome Assembly SNP, Illumina - BovineSNP50^®^, GWAS - significant markers at the genome wide association study.

^2 ^DTE - dairy tick experiment, BRM - Brahman, COM - Tropical Composite.

^3 ^Number of individuals tested.

^4 ^Frequency of the allele closer to a in the alphabet.

^5 ^Hardy-Weinberg equilibrium test p-value, ns - not significant.

**Figure 1:**
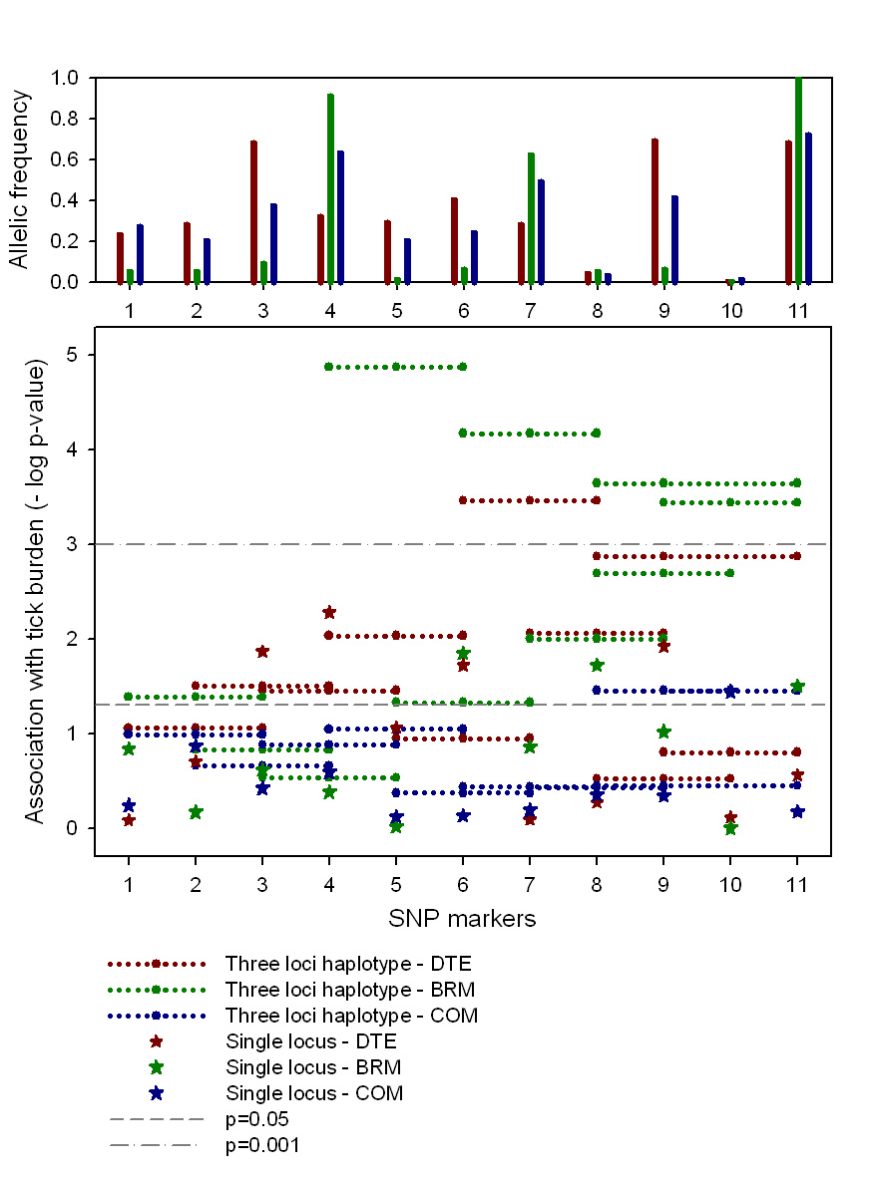
**SNP markers at the *ITGA11 *gene region: allelic frequency and analyses of marker association with tick burden in the three populations**. 1- rs29025980, 2- rs43616884, 3- rs29025985, 4- rs29025981, 5- rs41594962, 6-ss161109814, 7-ss161109807, 8- ss161109797, 9- rs29023635, 10- rs29023639, 11- rs29014770.

**Figure 2:**
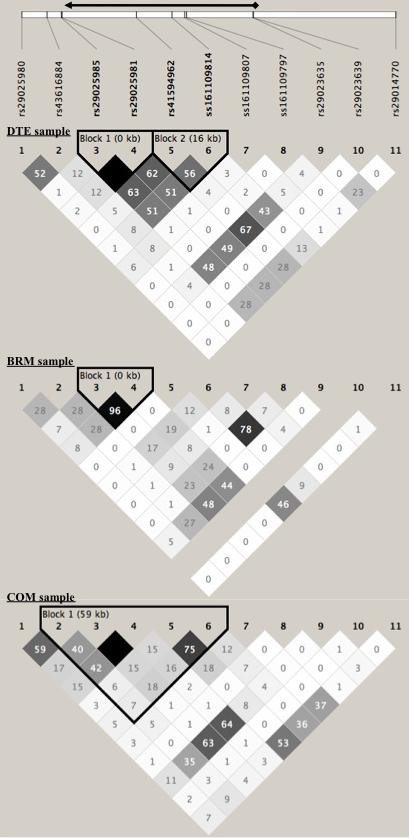
**Linkage Disequilibrium (LD), r^2 ^values, between all markers at the *ITGA11 *gene region**. The black arrow on the top represents the relative position of the *ITGA11 *gene.

There were significant allele and haplotype frequency differences between the DTE, BRM and COM animals. Allele frequencies for 9 of the 11 SNP were significantly different (*P *< 0.05) in the three groups of animals (Figure 1). The average *r^2 ^*between all SNP in the region was similar in all breeds, *r^2 ^*= 0.16 (DTE), 0.13 (BRM) and 0.15 (COM). The haplotype frequencies were also significantly (*P *< 0.05) different between groups. Nevertheless, there was some evidence of haplotype blocks in the same genetic region that contained *ITGA11 *(Figure 2 and additional file 2). The size of the haplotype block was smallest in BRM (< 1 kb) and largest in COM cattle (59 kb).

**Figure 3:**
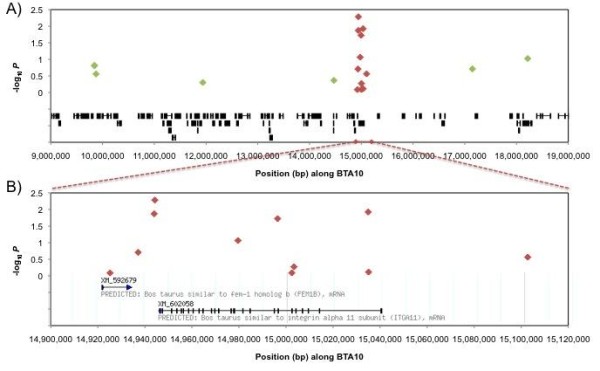
**Genetic map of SNP associations with tick burden in the dairy tick sample (DTE)**. **A) Associations with tick burden (-log P) of SNP markers between BTA10:9 Mb - BTA10:19 Mb and B) between BTA10:14.90 Mb - BTA10:15.12 Mb**. Markers in green were genotyped only in the DTE sample and markers in red were genotyped in all DTE, BRM and COM samples. The cattle RefSeq mRNA (Btau.4.0) located at the region are displayed at the base of each graph.

Four SNP were significantly associated with tick burden (*P *< 0.05) in the DTE sample, including both of the SNP used in the previous GWAS [20] (Table 2, Figure 1). The distribution of the SNP along BTA10 is shown in Figure 3. The most significant SNP were rs29025981 (*P *= 0.0052) and ss161109814 (*P *= 0.0188), the latter of these is from the *ITGA11 *gene. rs29025981 explained the most residual variance of *R^2 ^*= 0.9%.

**Table 2:** Significant SNP markers at the *ITGA11 *gene region associated with tick burden

Locus	Sample^1^	N^2^	p_0_^3^	R^2 4^	α^5^	SE^6^	p-value
rs29025985	DTE	812	0.68	0.0075	-0.131	0.053	0.0135

rs29025981	DTE	859	0.32	0.0091	0.147	0.053	0.0052

ss161109814	DTE	1037	0.40	0.0053	0.109	0.046	0.0188
	
	BRM	526	0.06	0.0115	-0.309	0.125	0.0140

ss161109797	BRM	526	0.05	0.0105	-0.332	0.141	0.0188

rs29023635	DTE	914	0.69	0.0069	-0.127	0.05	0.0119

rs29023639	COM	181	0.01	0.0244	-1.476	0.698	0.0359

rs29014770	BRM	544	0.99	0.0085	0.601	0.279	0.0317

^1 ^DTE - dairy tick experiment, BRM - Brahman, COM - Tropical Composite.

^2 ^Number of individuals tested.

^3 ^Frequency of the allele closer to A in the alphabet.

^4 ^Proportion of residual variance explained.

^5 ^Allele substitution effect in phenotypic standard deviations (tick count for the DTE and tick score for BRM and COM).

^6 ^Standard error of α.

Four of the 11 SNP genotyped in the Brahman and Tropical Composite cattle were significantly (*P *< 0.05) associated with tick scores (Table 2). Two of the SNP were from the genome assembly and two were from sequencing the *ITGA11 *gene. One of these, ss161109814, had been significantly (*P *< 0.05) associated with tick counts in the DTE sample. ss161109814 accounted for 1.2% of the residual variance (*R^2^*), another SNP (rs29023639) accounted for more but this SNP was significant in a small sample (COM) and was therefore possibly overestimated in amount of variance explained (Table 2). However, a different allele for ss161109814 was favourable for tick score in the Brahman and Tropical Composite sample (Table 2) compared to the DTE result.

In all samples of cattle there were highly significant (*P *< 0.001) associations between haplotypes using either a haplotype of 10 SNP that includes the *ITGA11 *gene or for several of the 3-locus haplotypes that are a subset of the 10 SNP (Table 3 and additional files 3 and 4). The 3-locus haplotypes that include SNP from the *ITGA11 *were significantly associated with tick burden in the DTE (n = 4) and in the BRM (n = 6) samples. Many of these associations remained significant after Bonferroni correction of the significance threshold. Although many of these highly significant associations were for relatively rare haplotypes, where no animal homozygous for the haplotype was found, in five of the 3-locus haplotypes that were highly significantly (P ≤ 0.0053) associated with tick burden there were two or more individuals that had two copies of the haplotype. Three of these comparisons were for taurine animals and two were for the BRM animals. Four of these haplotypes also showed some similarity of structure between samples - they show the allele '1' at the 8 and 9^th ^loci of the haplotypes in the BRM sample and the same alleles at those positions in the DTE sample. There was a low genotyping completion of locus 10 in the DTE sample, so haplotype analyses were run without that SNP in the DTE sample. The haplotype "h3" formed by the 8, 9 and 11^th ^loci was significantly associated with tick burden with its significance exceeding the Bonferroni correction threshold in the DTE and BRM samples. The favourable haplotype of these 3 loci (8,9,11) was common in BRM and rare in DTE cattle. In the DTE sample, the most significant 3-locus common haplotype ("6,7,8 h1") accounted for 1.25% of the residual variance (*R^2^*) (Table 3). In the BRM sample, the most significant 3-locus common haplotype ("8,9,10 h5") accounted for 1.83% of the residual variance (*R^2^*) (Table 3). The significant haplotypes that are relatively common (i.e. with more than 2 individuals homozygous for the rarer haplotype) in our samples had haplotype substitution effects of the same, negative, sign in all cases.

**Table 3:** Significant haplotypes of markers at the *ITGA11 *gene region associated with tick burden

SNP^1^	Code	Sample^2^	N0^3^	N1	N2	R^2 4^	α^5^	SE^6^	p-value
10snp h10	110111111 - 0	DTE (48)	605	17	0	0.0146	-0.738	0.243	0.0025

10snp h19	111000111 - 0	DTE (48)	582	38	2	0.0131	-0.432	0.151	0.0043

10snp h23	111010101 - 0	BRM (24)	419	4	0	0.0422	2.119	0.492	2 × 10^-5^^

4,5,6 h7	- - - 010 - - - - -	DTE (8)	808	23	1	0.0082	-0.507	0.194	0.0092
		
		BRM (5)	477	8	0	0.0386	1.542	0.350	1 × 10^-5^^

6,7,8 h1	- - - - - 001 - - -	DTE (7)	891	124	10	0.0125	-0.297	0.083	0.0003^

7,8,9 h2	- - - - - - 011 - -	DTE (7)	734	156	0	0.0077	-0.231	0.088	0.0087

7,8,9 h7	- - - - - - 101 - -	BRM (5)	476	42	2	0.0128	0.380	0.147	0.0099^

8,9,10 h2	- - - - - - - 011 -	BRM (3)	477	42	2	0.0128	0.381	0.147	0.0097^

8,9,10 h5	- - - - - - - 111 -	BRM (3)	6	98	417	0.0183	-0.310	0.100	0.0020^

8,9,11 h2	- - - - - - - 10 - 1	BRM (5)	513	5	0	0.0261	1.651	0.444	0.0002^

8,9,11 h3	- - - - - - - 11 - 0	DTE (6)	538	332	8	0.0117	-0.212	0.066	0.0013^
		
		BRM (5)	8	102	408	0.0150	-0.269	0.096	0.0053^

9,10,11 h2	- - - - - - - - 011	BRM (5)	537	5	0	0.0233	1.596	0.444	0.0004^

^1 ^SNP markers used to generate the haplotypes (4- rs29025981, 5- rs41594962, 6- ss161109814, 7- ss161109807, 8- ss161109797, 9- rs29023635, 10- rs29023639, 11- rs29014770) and haplotype number.

^2 ^DTE - dairy tick experiment, BRM - Brahman and number of haplotypes reconstructed.

^3 ^N0 number of animals with zero copies of the haplotype, N1 number of animals with one copy of the haplotype, N2 number of animals with two copies of the haplotype.

^4 ^Proportion of residual variance explained by the common haplotype.

^5 ^Haplotype substitution effect in phenotypic standard deviations (tick count for the DTE and tick score for BRM).

^6 ^Standard error of α.

^ Significant after Bonferroni correction.

## Discussion

In this study we have confirmed that there is a QTL affecting tick burden on BTA10. There were significant (*P *< 0.05) single marker associations to tick burden in DTE, BRM and COM animals which were more significant (*P *< 0.001) when these markers were analysed as haplotypes. This included haplotypes that incorporated DNA variation from the *ITGA11 *gene. One of the SNP was significant (*P *< 0.05) in both taurine and zebu animals but the favourable allele was different in effect size and direction. A different favourable allele in two populations is likely to be due to either a spurious association between the trait and the genotypes or due to different patterns of linkage disequilibrium in the two samples between markers and the causative mutation.

Where populations are similar and genetically closely related, different favourable alleles in two samples may be spurious. However, the ancestors of zebu and taurine cattle were separated for more than half a million years before domestication [23], so their population haplotypes are not expected to be similar. Furthermore, an analysis of LD and haplotype structure in the breed types showed that allele and haplotype frequencies were very different between these cattle types in this genetic region. This suggests that LD relationships would likely be different between SNP and that the difference in favourable allele for the SNP ss161109814 could be due to differences in LD and not due to spurious association. Indeed, it is possible, with such a large evolutionary distance from the common ancestor to these two separated groups, for the causative mutations to be different in these two breed types. In the haplotype analyses, for the relatively common haplotypes that were highly significant in the DTE and BRM cattle samples, haplotypes with the '1' allele at SNP positions 8, 9, 10 and 11 were significant and showed a similar favourable effect, of -0.21 and -0.27 phenotypic standard deviations respectively (Table 3). More importantly, the favourable form of the haplotype was common in the BRM sample but rare in the DTE sample, consistent with the relatively low tick numbers on BRM cattle and high tick numbers of DTE cattle [8-10].

The amount of the variation explained by the markers (*R^2^*), estimated by single SNP or through haplotypes, is approximately 1% of the residual variance in moderate to large samples. Some of the rarer haplotypes have effect sizes of > 1 phenotypic standard deviation, but as these involve a small number of heterozygous genotypes these effect sizes have been discounted as likely due to sampling effects. Haplotypes are more likely to reflect the size of effect of a causal mutation than most single markers that are in LD to the causal mutation(s), because recombination takes longer to degrade the relationship between a causal allele and a haplotype than to a single SNP. Further research might determine whether the effect of a causative mutation is large, as shown for some of the haplotypes or, more likely, are relatively small as shown by the more frequent haplotypes. Mutations accounting for a small proportion of the genetic variation (*R^2^*) are commonly identified in QTL mapping studies in cattle and other species. However, it is not yet definitively shown whether causative alleles for these smaller QTL are common variants that have small effects or are due to a large number of very rare QTL each of relatively large effect [24]. Further discovery of new genetic variation will be needed to identify such putative causative mutations which appear to be located near the *ITGA11 *gene.

The adaptive immune system has long been shown to be important in tick resistance in cattle [25,26], and *ITGA11 *is neither a part of the adaptive immune system nor known to be part of the innate immune system. In this region the nearest genes that are part of the immune system are *PIAS1 *(*protein inhibitor of activated STAT, 1*, BTA10:14,721,762) and *ANP32A *(*acidic (leucine rich) nuclear phosphoprotein 32 family, member A*, BTA10:15,806,500). In the previous GWAS [20], SNP near those genes were not significantly associated with tick burden. Moreover, the significant SNP in this study are more than 200 kb from *PIAS1 *and nearly 1 Mb from *ANP32A*. Genotyping of SNP in this study over the 8.4 Mb of this region of BTA10, which includes these genes, failed to find a signal of association in other parts of the chromosomal segment at the density we used. Our estimates of LD in this region in these samples show low values, consistent with other studies of samples with multiple breeds [27]. Although we cannot categorically reject the influence of those genes, it is unlikely that the significant associations that we found is due to LD to those genes of the adaptive immune system.

Although *ITGA11 *is not an obvious positional candidate gene for tick burdens, because its biological role appears to be mainly in the control of cellular adhesion and migration, it cannot be rejected completely based on its function. Coelho et al. [28] identified *ITGA11 *as an interferon-inducible gene in human fibroblasts. It is possible that *ITGA11 *may play a role in modulating cellular immune responses, by influencing the recruitment and adhesion of immune cells at sites of infection, or ectoparasite infestation. Moreover, the integrity and composition of the dermis may play a role in an animal's defences against ticks. Integrins specifically interact with collagen. Experiments aimed at identifying genes that were differentially expressed in cattle with different tick resistance phenotypes, found evidence that collagen and other extracellular matrix genes were differentially expressed in the skin of cattle that are more resistant to tick infestation [29,30].

## Conclusions

Our analyses confirmed that there is a QTL affecting tick burden on BTA10. Significant common haplotypes were found that accounted for 1% of the residual variance and these haplotypes incorporated DNA variation from the *ITGA11 *gene. Whether the effects observed are due to variation in *ITGA11 *itself or are due to cis-effects of variation near *ITGA11 *regulating other genes will require in-depth study of gene expression and function. Further analyses of SNP and other kinds of DNA variation in this region would be a first step toward identifying the causal alleles and elucidating the biological mechanism involved.

## Methods

The analysis in the study follows a specific order. First, a selection of 32 putative SNP from BTA10 was genotyped in a collection of dairy taurine cattle, the dairy tick experiment (DTE). Fifteen of these putative SNP were monomorphic, which reduced the total available SNP to 17. Of these 17, 2 SNP were significant in the initial GWAS [20]. Second, 11 of these SNP were then genotyped in Brahman (BRM) and tropical composite (COM) beef cattle to extend and confirm the associations. As part of the analysis, single SNP as well as haplotype analyses were performed.

### Tick phenotypes

The animals, tick phenotypes and DNA samples were described previously [8,9]. In brief, the number of ticks on these animals (tick burden) was estimated in one of two ways. The DTE sample had field tick counts of individual ticks in the size range of 4.5 - 8 mm in diameter which represents mature ticks that will fall off the animal in the next 24 hours [4]. Ticks were counted on one side of the animal. The BRM and COM sample had tick burden estimates using tick scores. Tick scores are rapid estimates of tick burden of ticks that are > 4.5 mm in diameter. The tick scores are on a 0 - 5 scale where 0 is no ticks, 1 is ≤ 10 ticks, 2 is 11 - 30 ticks, 3 is 31 - 80 ticks, 4 is 81 - 150 ticks, and 5 > 150 ticks. Although tick scores are underestimates, are less accurate and they are also less informative, nevertheless there is a high genetic correlation between the two measurements [31]. Due to the unavailability of beef cattle with tick counts, we were constrained to using existing tick scores to confirm the associations that we had observed in the DTE.

### Animal samples

In this experiment we used DNA samples from 1,055 DTE cattle that had ≥ 2 tick counts. Animals were described in detail elsewhere [8]. In brief, the DTE cattle were from 16 properties across the tick zone in tropical and sub-tropical northeastern Australia. The sample consisted of animals of the Australian Red breed (AUR, n = 196), the Brown Swiss breed and its crosses (BSWX, n = 126), the Channel Isle breeds and their crosses (CHA, n = 119), the Holstein breed and its crosses (HOLX, n = 187), composite taurine cattle (MIXT, n = 424) and composite taurine cattle with at least one grandparent of zebu ancestry (ZEBX, n = 3). The previously published principal component analysis of genotypes of these taurine dairy cattle of pure and mixed ancestry could not put breeds into separate clusters or distinguish crossbreds from purebreds [32] so crossbreds were lumped with appropriate purebreds on the basis of known ancestry as previously described [8]. The average field tick counts for these animals was 47.1 ticks per side or an average ln(ticks+1) of 3.03 ± 1.29 (s.d.) [8].

To confirm the associations we used 557 BRM and 216 COM animals that together form 773 Cooperative Research Centre (CRC2) animals with tick score data [33]. These cattle have been extensively described in a series of open access articles, see [33]. The COM animals were 50% *Bos taurus indicus*, African sanga or other tropically adapted *Bos taurus *and 50% non-adapted *Bos taurus taurus*. The animals with tick scores were bred on the Belmont and Swans Lagoon research stations for the summer of 2003/4 (December - February) and all were females with mean age of 34 months [9]. The average field tick score for BRM was 0.75 (s.d. = 0.74) and for COM was 2.26 (s.d. = 0.98) [9]. The adjustment of the phenotypic data was performed as previously specified [33].

### Analysis of tick data

The association between each SNP and tick burden was assessed by a regression analysis of a residual phenotype on numbers of copies of a particular allele. To obtain the residual phenotype, trait values were fitted in a mixed model using the ASReml software [34] as follows: trait ~ mean + fixed effects + animal + error, with animal and error fitted as random effects. For the DTE sample, the fixed effects of property, season and breed type were modelled as main fixed effects, where season included the identity of the counter and all tick counts of an animal were included indexed by season. All available pedigree information (sire, dam, grandsire and maternal grandsire identities) was included in the model. The residual effect of the animal was extracted for SNP regression analysis. For the CRC2 sample, the trait tick score was modelled with the fixed effects of breed, herd of origin, cohort, calving month and their first-degree interactions. Three generations of pedigree information was available. Residual trait values were extracted and used in SNP regression analyses. These models do not include the effects of DNA polymorphisms.

### SNP markers and genotypes

A panel of SNP markers was genotyped over an 8.4 Mb region of BTA10. The panel included 2 SNP (rs29025985 and rs29025981) significantly associated to tick burden in the GWAS [20], 9 SNP from the Baylor College of Medicine (BCM) interbreed database, 2 SNP from the Illumina^® ^BovineSNP50 and 16 SNP from the BCM bovine genome assembly database ftp://ftp.hgsc.bcm.tmc.edu/pub/data/Btaurus/snp/Btau20050310/. To add to the number of SNP, and based on the SNP that had been significant in previous studies (rs29025985, rs29025981 and rs29025982), we sequenced PCR products of exons of the *ITGA11 *gene and the intronic sequence surrounding these exons. The cDNA sequence for *ITGA11 *(Genbank XM_602058.3) was compared to the cattle genome sequence using BLAST [35] to determine the splice sites of the gene. Primers to amplify exons 6 to 9 were designed, which correspond to the I-Domain of the protein [36]. Forward and reverse DNA sequence were obtained from four animals each of four breeds (Angus, Shorthorn, Holstein and Brahman). SNP were described using standard nomenclature [37]. SNP were submitted to dbSNP http://www.ncbi.nlm.nih.gov/snp and assigned identifiers (Additional file 1). SNP were genotyped using either GoldenGate^® ^(Illumina Inc., Hayward, California), SNPlex™ or TaqMan^® ^SNP Genotyping Assays (Applied Biosystems, Foster City, California) following the manufacturer's instructions with scoring performed by two individuals before genotypes were merged with phenotypes.

### Analyses of genotypic data

The SNP genotypes were tested for Hardy-Weinberg equilibrium (HWE) within breed type using PLINK 1.05 [[38], http://pngu.mgh.harvard.edu/purcell/plink/]. The linkage disequilibrium (LD) between SNP was estimated using Haploview 4.1 [39] for DTE, BRM and COM individually. The haplotype block structure was obtained using the confidence interval method [40] implemented in Haploview 4.1. For association analyses, haplotypes were obtained using PHASE 2.1.1 [41,42]. We applied the PHASE algorithm five times for each set of SNP on animals without missing genotypes for the SNP in the haplotype. We also allowed PHASE to interpolate missing data and then evaluated the associations (Additional file 3). We evaluated a haplotype of all available SNP as well as a "sliding window" of haplotypes of three adjacent SNP (3-locus haplotype), sliding one SNP at a time across the genomic region. Each 3-locus haplotype was named for the markers used to generate the haplotype plus the number of the haplotype generated by these markers; e.g. haplotype 5,6,7 h1 was generated using the markers 5, 6 and 7 and this is the first haplotype (h1) of this set of markers. All haplotypes identified in this study are listed in additional file 4.

Association between each SNP or haplotype was evaluated by regression of the residual tick count or score on the number of copies of a reference allele. Allele associations were performed one SNP at a time. Significance was evaluated using a t-test of the slope of the regression over its standard error for each marker individually. For each haplotype, the individuals were scored for the number of copies of the haplotype they possessed, each haplotype was considered an independent event, analogous to analyses of DNA microsatellites [43], and the residual tick counts or scores were regressed on the number of copies of haplotypes analysed one at a time. A t-test was calculated by dividing the regression coefficient by its standard error. For association analyses of haplotypes the significance threshold was adjusted for multiple testing by Bonferroni correction, dividing the nominal 5% significance threshold by the number of haplotypes inferred by PHASE for a set of SNP [43].

## Authors' contributions

LRPN and WB planned the experiments and wrote the manuscript. LRPN analyzed the genotypic data, RJB and BEH collected DNA samples. LRPN, RJB and BEH genotyped the animals. LRPN identified SNP by DNA sequencing, KCP defined the beef cattle phenotypes. All authors read and approved the final manuscript.

## Supplementary Material

Additional file 1 **Description of discovered SNP in the *ITGA11 *gene**. Table describing the discovered SNP Btau 4.0 positions and dbSNP ss numbers. Click here for file

Additional file 2 **Linkage disequilibrium (LD) between markers at the *ITGA11 *gene region**. Linkage disequilibrium (LD) between markers at the *ITGA11 *gene region. Click here for file

Additional file 3 **Eleven loci haplotype association with tick burden: interpolated missing data**. Eleven loci haplotype association with tick burden using the interpolated missing data. Click here for file

Additional file 4 **Description of the haplotypes reconstructed using 10 loci and the 3-locus sliding window**. Description of the haplotypes reconstructed using 10 loci and the 3-locus sliding window. Click here for file

## References

[B1] Minjauw B McLeod A Tick-borne diseases and poverty. The impact of ticks and tick-borne diseases on the livelihoods of small-scale and marginal livestock owners in India and eastern and southern Africa 2003 Research Report, DFID Animal Health Programme, Centre for Tropical Veterinary Medicine, University of Edinburgh, UK

[B2] Jongejan F Uilenberg G The global importance of ticks Parasitology 2004 129 S3 S14 10.1017/S0031182004005967 15938502

[B3] deCastro JJ Sustainable tick and tickborne disease control in Livestock improvement in developing countries Veterinary Parasitology 1997 71 2-3 77 97 10.1016/S0304-4017(97)00033-2 9261972

[B4] Wharton RH Utech KBW Turner HG Resistance to cattle tick, Boophilus-microplus in a heard of Australian Illawarra Shorthorn cattle - its assessment and heritability Australian Journal of Agricultural Research 1970 21 1 163 10.1071/AR9700163

[B5] Mackinnon MJ Meyer K Hetzel DJS Genetic-variation and covariation for growth, parasite resistance and heat tolerance in tropical cattle Livestock Production Science 1991 27 2-3 105 122 10.1016/0301-6226(91)90090-D

[B6] Burrow HM Variances and covariances between productive and adaptive traits and temperament in a composite breed of tropical beef cattle Livestock Production Science 2001 70 3 213 233 10.1016/S0301-6226(01)00178-6

[B7] Prayaga KC Henshall JM Adaptability in tropical beef cattle: genetic parameters of growth, adaptive and temperament traits in a crossbred population Australian Journal of Experimental Agriculture 2005 45 7-8 971 983 10.1071/EA05045

[B8] Turner LB Harrison BE Bunch RJ Porto Neto LR Li YT Barendse W A genome wide association study of tick burden and milk composition in cattle Animal Production Science 2010 50 235 245 10.1071/AN09135

[B9] Prayaga KC Corbet NJ Johnston DJ Wolcott ML Fordyce G Burrow HM Genetics of adaptive traits in heifers and their relationship to growth, pubertal and carcass traits in two tropical beef cattle genotypes Animal Production Science 2009 49 5-6 413 425 10.1071/EA08247

[B10] Utech KBW Wharton RH Kerr JD Resistance to Boophilus-microplus (Canestrini) in different breeds of cattle Australian Journal of Agricultural Research 1978 29 4 885 895 10.1071/AR9780885

[B11] Frisch JE O'Neill CJ Comparative evaluation of beef cattle breeds of African, European and Indian origins. 2. Resistance to cattle ticks and gastrointestinal nematodes Animal Science 1998 67 39 48

[B12] da Silva AM de Alencar MM de Almeida LC Regitano de Sena Oliveira MC Barioni W Artificial infestation of Boophilus microplus in beef cattle heifers of four genetic groups Genetics and Molecular Biology 2007 30 4 1150 1155

[B13] Stear MJ Hetzel DJ Brown SC Gershwin LJ Mackinnon MJ Nicholas FW The relationships among ectoparasite and endoparasite levels, class I antigens of the bovine major histocompatibility system, immunoglobulin E levels and weight gain Veterinary Parasitology 1990 34 4 303 321 10.1016/0304-4017(90)90077-O 2316176

[B14] Stear MJ Nicholas FW Brown SC Holroyd RG Class I antigens of the bovine major histocompatibility system and resistance to the cattle tick (Boophilus microplus) assessed in three different seasons Veterinary Parasitology 1989 31 3-4 303 315 10.1016/0304-4017(89)90080-0 2763449

[B15] Acosta-Rodrigez R Alonso-Morales R Balladares S Flores-Aguilar H Garcia-Vazquez Z Gorodezky C Analysis of BoLA class II microsatellites in cattle infested with Boophilus microplus ticks: class II is probably associated with susceptibility Veterinary Parasitology 2005 127 3-4 313 321 10.1016/j.vetpar.2004.10.007 15710532

[B16] Martinez ML Machado MA Nascimento CS Silva MV Teodoro RL Furlong J Prata MC Campos AL Guimaraes MF Azevedo AL Association of BoLA-DRB3.2 alleles with tick (Boophilus microplus) resistance in cattle Genetics and Molecular Research 2006 5 3 513 524 17117367

[B17] Untalan PM Pruett JH Steelman CD Association of the bovine leukocyte antigen major histocompatibility complex class II DRB3*4401 allele with host resistance to the Lone Star tick, Amblyomma americanum Veterinary Parasitology 2007 145 1-2 190 195 10.1016/j.vetpar.2006.12.003 17208379

[B18] Regitano LCA Ibelli AMG Gasparin G Miyata M Azevedo ALS Coutinho LL Teodoro RL Machado MA Silva M Nakata LC Pinard MH, Gay C, Pastoret PP, Dodet B On the Search for Markers of Tick Resistance in Bovines Animal Genomics for Animal Health 2008 132 Basel: Karger 225 230 full_text 10.1159/000317164 18817306

[B19] Gasparin G Miyata M Coutinho LL Martinez ML Teodoro RL Furlong J Machado MA Silva M Sonstegard TS Regitano LCA Mapping of quantitative trait loci controlling tick [Riphicephalus (Boophilus) microplus] resistance on bovine chromosomes 5, 7 and 14 Animal Genetics 2007 38 5 453 459 10.1111/j.1365-2052.2007.01634.x 17894560

[B20] Barendse W Assessing tick resistance in a bovine animal for selecting cattle for tick resistance by providing a nucleic acid from the bovine animal and assaying for the occurrence of a single nucleotide polymorphism (SNP) Patent application WO2007051248-A1 2007 1 146

[B21] Elsik CG Tellam RL Worley KC Gibbs RA Muzny DM Weinstock GM Adelson DL Eichler EE Elnitski L Guigo R The genome sequence of taurine cattle: a window to ruminant biology and evolution Science 2009 324 5926 522 528 10.1126/science.1169588 19390049 PMC2943200

[B22] Porto Neto LR Barendse W Automated SNP calling overestimates the number of SNP in the bovine genome Proceedings of the Conference of the International Society for Animal Genetics Amsterdam 2008 1 3 http://www.isag.org.uk/conferences_past.asp checked at 11/06/2010.

[B23] MacHugh DE Shriver MD Loftus RT Cunningham P Bradley DG Microsatellite DNA variation and the evolution, domestication and phylogeography of taurine and Zebu cattle (Bos taurus and Bos indicus) Genetics 1997 146 3 1071 1086 9215909 10.1093/genetics/146.3.1071 PMC1208036

[B24] Pritchard JK Are rare variants responsible for susceptibility to complex diseases? American Journal of Human Genetics 2001 69 1 124 137 10.1086/321272 11404818 PMC1226027

[B25] Willadsen P Immunity to ticks Advances in Parasitology 1980 18 293 311 full_text 7435340 10.1016/s0065-308x(08)60402-9

[B26] Inokuma H Kerlin RL Kemp DH Willadsen P Effects of cattle tick (Boophilus-microplus) infestation on the bovine immune-system Veterinary Parasitology 1993 47 1-2 107 118 10.1016/0304-4017(93)90181-L 8493757 PMC7131769

[B27] Gibbs RA Taylor JF Van Tassell CP Barendse W Eversole KA Gill CA Green RD Hamernik DL Kappes SM Lien S Genome-wide survey of SNP variation uncovers the genetic structure of cattle breeds Science 2009 324 5926 528 532 10.1126/science.1167936 19390050 PMC2735092

[B28] Coelho LFL Mota BEF Sales PCM Marques JT de Oliveira JG Bonjardim CA Ferreira PCP Kroon EG Integrin alpha 11 is a novel type I interferon stimulated gene Cytokine 2006 33 6 352 361 10.1016/j.cyto.2006.03.007 16697656

[B29] Kongsuwan K Piper EK Bagnall NH Ryan K Moolhuijzen P Bellgard M Lew A Jackson L Jonsson NN Pinard MH, Gay C, Pastoret PP, Dodet B Identification of Genes Involved with Tick Infestation in Bos taurus and Bos indicus Animal Genomics for Animal Health 2008 132 Basel: Karger 77 88 full_text 10.1159/000317146 18817288

[B30] Wang YH Reverter A Kemp D McWilliam SM Ingham A Davis CK Moore RJ Lehnert SA Gene expression profiling of Hereford Shorthorn cattle following challenge with Boophilus microplus tick larvae Australian Journal of Experimental Agriculture 2007 47 12 1397 1407 10.1071/EA07012

[B31] Fraga AB Alencar MMd Figueiredo LAd Razook AG Cyrillo JNdSG Analise de fatores geneticos e ambientais que afetam a infestacao de femeas bovinas da raca Caracu por carrapatos (Boophilus microplus) Revista Brasileira de Zootecnia 2003 32 1578 1586 10.1590/S1516-35982003000700006

[B32] Barendse W Harrison BE Bunch RJ Thomas MB Turner LB Genome wide signatures of positive selection: The comparison of independent samples and identification of regions associated to traits BMC Genomics 2009 10 178 10.1186/1471-2164-10-178 19393047 PMC2681478

[B33] Barwick SA Johnston DJ Burrow HM Holroyd RG Fordyce G Wolcott ML Sim WD Sullivan MT Genetics of heifer performance in 'wet' and 'dry' seasons and their relationships with steer performance in two tropical beef genotypes Animal Production Science 2009 49 5-6 367 382 10.1071/EA08273

[B34] Gilmour AR Gogel BJ Cullis BR Welham SJ Thompson R ASReml User Guide Release 1.0 2002 VSN International Ltd, Hemel Hempstead, HP1 1ES, UK

[B35] Altschul SF Gish W Miller W Myers EW Lipman DJ Basic Local Aligment Search Tool Journal of Molecular Biology 1990 215 3 403 410 2231712 10.1016/S0022-2836(05)80360-2

[B36] Takada Y Ye XJ Simon S The integrins Genome Biology 2007 8 5 10.1186/gb-2007-8-5-215 17543136 PMC1929136

[B37] den Dunnen JT Antonarakis SE Mutation nomenclature extensions and suggestions to describe complex mutations: A discussion Human Mutation 2000 15 1 7 12 10.1002/(SICI)1098-1004(200001)15:1<7::AID-HUMU4>3.0.CO;2-N 10612815

[B38] Purcell S Neale B Todd-Brown K Thomas L Ferreira MAR Bender D Maller J Sklar P de Bakker PIW Daly MJ PLINK: A tool set for whole-genome association and population-based linkage analyses American Journal of Human Genetics 2007 81 3 559 575 10.1086/519795 17701901 PMC1950838

[B39] Barrett JC Fry B Maller J Daly MJ Haploview: analysis and visualization of LD and haplotype maps Bioinformatics 2005 21 2 263 265 10.1093/bioinformatics/bth457 15297300

[B40] Gabriel SB Schaffner SF Nguyen H Moore JM Roy J Blumenstiel B Higgins J DeFelice M Lochner A Faggart M The structure of haplotype blocks in the human genome Science 2002 296 5576 2225 2229 10.1126/science.1069424 12029063

[B41] Stephens M Smith NJ Donnelly P A new statistical method for haplotype reconstruction from population data American Journal of Human Genetics 2001 68 4 978 989 10.1086/319501 11254454 PMC1275651

[B42] Stephens M Scheet P Accounting for decay of linkage disequilibrium in haplotype inference and missing-data imputation American Journal of Human Genetics 2005 76 3 449 462 10.1086/428594 15700229 PMC1196397

[B43] Pritchard JK Rosenberg NA Use of unlinked genetic markers to detect population stratification in association studies American Journal of Human Genetics 1999 65 1 220 228 10.1086/302449 10364535 PMC1378093

