# Supplementary material for: Haplotypes that include the integrin alpha 11 gene are associated with tick burden in cattle
Source: BMC Genet. 2010 Jun 21;11:55. doi: 10.1186/1471-2156-11-55 (PMC2905322; doi:10.1186/1471-2156-11-55)
Supplement: Additional file 1 — Description of discovered SNP in the ITGA11 gene. Table describing the discovered SNP Btau 4.0 positions and dbSNP ss numbers. [file 1471-2156-11-55-S1.PDF]

ADDITIONAL FILES

Table S1. Description of discovered SNP in the *ITGA11* gene.

SNP_identifier	Btau4.0 (Chr10)	dbSNP
AAFC03010801.1:g.26278A>G	15003812	ss161109791
AAFC03010801.1:g.26461C>T	15003629	ss161109792
AAFC03010801.1:g.26500C>T	15003590	ss161109793
AAFC03010801.1:g.26512A>G	15003578	ss161109794
AAFC03010801.1:g.26561C>G	15003529	ss161109795
AAFC03010801.1:g.26613A>C	15003477	ss161109796
AAFC03010801.1:g.26699C>T	15003391	ss161109797
AAFC03010801.1:g.26731A>G	15003359	ss161109798
AAFC03010801.1:g.26756C>G	15003334	ss161109799
AAFC03010801.1:g.26788T>G	15003302	ss161109800
AAFC03010801.1:g.26832C>T	15003258	ss161109801
AAFC03010801.1:g.26844A>G	15003246	ss161109802
AAFC03010801.1:g.26936A>C	15003154	ss161109803
AAFC03010801.1:g.26944C>T	15003146	ss161109804
AAFC03010801.1:g.26991A>G	15003099	ss161109805
AAFC03010801.1:g.27136C>T	15002954	ss161109806
AAFC03010801.1:g.27594C>T	15002496	ss161109807
AAFC03010801.1:g.27656C>T	15002434	ss161109808
AAFC03010801.1:g.27737C>G	15002353	ss161109809
AAFC03010801.1:g.28025A>G	15002065	ss161109810
AAFC03010801.1:g.28044A>G	15002046	ss161109811
AAFC03010801.1:g.28052A>G	15002038	ss161109812
AAFC03010801.1:g.33276A>G	14996814	ss161109813
AAFC03010801.1:g.33650C>T	14996440	ss161109814
AAFC03010801.1:g.33711A>G	14996379	ss161109815
AAFC03010801.1:g.33718C>T	14996372	ss161109816
