# Supplementary material for: Haplotypes that include the integrin alpha 11 gene are associated with tick burden in cattle
Source: BMC Genet. 2010 Jun 21;11:55. doi: 10.1186/1471-2156-11-55 (PMC2905322; doi:10.1186/1471-2156-11-55)
Supplement: Additional file 2 — Linkage disequilibrium (LD) between markers at the ITGA11 gene region. Linkage disequilibrium (LD) between markers at the ITGA11 gene region. [file 1471-2156-11-55-S2.PDF]

Table S2. Linkage disequilibrium (LD) between markers at the *ITGA11* gene region.

		DTE[1]		BRM		COM	
SNP_1	SNP_2	*D'*	r^2	*D'*	r^2	*D'*	r^2
rs29025980	rs43616884	0.801	0.522	0.584	0.285	0.981	0.593
rs29025980	rs29025985	0.373	0.019	0.370	0.071	0.560	0.173
rs29025980	rs29025981	0.381	0.020	0.423	0.088	0.501	0.158
rs29025980	rs41594962	0.273	0.009	1.000	0.000	0.561	0.031
rs29025980	ss161109814	0.293	0.017	0.064	0.004	0.656	0.053
rs29025980	ss161109807	0.297	0.065	0.068	0.000	0.281	0.033
rs29025980	ss161109797	0.788	0.007	0.090	0.007	1.000	0.012
rs29025980	rs29023635	0.257	0.009	0.248	0.050	0.454	0.115
rs29025980	rs29023639	1.000	0.000	-	-	1.000	0.020
rs29025980	rs29014770	0.076	0.004	0.307	0.000	0.668	0.070
rs43616884	rs29025985	0.816	0.122	0.747	0.284	1.000	0.405
rs43616884	rs29025981	0.825	0.125	0.758	0.280	1.000	0.421
rs43616884	rs41594962	0.581	0.052	1.000	0.000	1.000	0.063
rs43616884	ss161109814	0.581	0.089	0.122	0.013	1.000	0.079
rs43616884	ss161109807	0.288	0.081	0.423	0.015	0.443	0.053
rs43616884	ss161109797	1.000	0.015	0.117	0.012	1.000	0.008
rs43616884	rs29023635	0.529	0.048	0.612	0.276	0.974	0.355
rs43616884	rs29023639	1.000	0.000	-	-	1.000	0.032
rs43616884	rs29014770	0.184	0.006	0.393	0.000	1.000	0.099
rs29025985	rs29025981	1.000	1.000	1.000	0.962	1.000	1.000
rs29025985	rs41594962	0.881	0.630	0.759	0.001	1.000	0.157
rs29025985	ss161109814	0.844	0.514	0.569	0.174	0.953	0.187
rs29025985	ss161109807	0.315	0.018	0.737	0.091	0.152	0.013
rs29025985	ss161109797	0.285	0.006	0.752	0.234	0.532	0.011
rs29025985	rs29023635	0.750	0.483	0.834	0.487	0.866	0.639
rs29025985	rs29023639	1.000	0.003	-	-	1.000	0.016
rs29025985	rs29014770	0.560	0.282	0.150	0.003	0.414	0.043
rs29025981	rs41594962	0.880	0.629	0.727	0.000	1.000	0.151
rs29025981	ss161109814	0.847	0.518	0.608	0.191	0.908	0.165
rs29025981	ss161109807	0.330	0.019	0.685	0.080	0.198	0.023
rs29025981	ss161109797	0.287	0.006	0.788	0.243	0.498	0.012
rs29025981	rs29023635	0.751	0.491	0.821	0.445	0.865	0.644
rs29025981	rs29023639	1.000	0.002	-	-	1.000	0.014
rs29025981	rs29014770	0.561	0.285	0.153	0.003	0.412	0.039
rs41594962	ss161109814	0.987	0.562	1.000	0.129	1.000	0.752
rs41594962	ss161109807	0.513	0.04	1.000	0.012	0.864	0.183
rs41594962	ss161109797	0.143	0.002	0.040	0.000	0.257	0.007
rs41594962	rs29023635	0.855	0.674	0.001	0.000	0.701	0.085
rs41594962	rs29023639	1.000	0.002	-	-	1.000	0.002
rs41594962	rs29014770	0.559	0.284	0.873	0.467	0.920	0.532
ss161109814	ss161109807	0.234	0.030	0.923	0.083	0.636	0.129
ss161109814	ss161109797	0.661	0.024	1.000	0.787	0.861	0.070
ss161109814	rs29023635	0.824	0.430	0.032	0.001	0.438	0.043

ss161109814	rs29023639	1.000	0.002	-	-	1.000	0.002
ss161109814	rs29014770	0.461	0.136	0.659	0.093	0.658	0.362
ss161109807	ss161109797	0.150	0.002	1.000	0.075	0.317	0.003
ss161109807	rs29023635	0.539	0.050	0.652	0.046	0.084	0.005
ss161109807	rs29023639	1.000	0.006	-	-	0.017	0.000
ss161109807	rs29014770	0.279	0.014	0.526	0.006	1.000	0.373
ss161109797	rs29023635	0.707	0.044	0.709	0.002	0.437	0.004
ss161109797	rs29023639	1.000	0.000	-	-	1.000	0.000
ss161109797	rs29014770	1.000	0.018	0.240	0.000	1.000	0.012
rs29023635	rs29023639	1.000	0.002	-	-	1.000	0.012
rs29023635	rs29014770	0.483	0.230	0.336	0.019	0.337	0.030
rs29023639	rs29014770	1.000	0.001	-	-	1.000	0.002

[1] DTE – dairy tick experiment, BRM – Brahman, COM – Tropical Composite.
