# Supplementary material for: Haplotypes that include the integrin alpha 11 gene are associated with tick burden in cattle
Source: BMC Genet. 2010 Jun 21;11:55. doi: 10.1186/1471-2156-11-55 (PMC2905322; doi:10.1186/1471-2156-11-55)
Supplement: Additional file 3 — Eleven loci haplotype association with tick burden: interpolated missing data. Eleven loci haplotype association with tick burden using the interpolated missing data. [file 1471-2156-11-55-S3.PDF]

Table S3. Eleven loci haplotype association with tick burden: interpolated missing data.

Haplotype	Code	Sample[1]	N0[2]	N1	N2	R^2 [3]	α[4]	SE[5]	p-value
h9	11011111110	DTE (52)	1032	23	0	0.0130	-0.779	0.209	0.0002^
h14	11101001010		1033	22	0	0.0069	-0.578	0.214	0.0071
h20	11101111010		1043	12	0	0.0037	-0.575	0.289	0.0470
h30	11100011110		994	58	3	0.0057	-0.301	0.122	0.0142
h35	10011111010		963	87	5	0.0043	0.215	0.101	0.0327
h45	01100011110		1048	7	0	0.0081	-1.103	0.377	0.0035
h18	11101010110	BRM (27)	548	5	0	0.0238	1.627	0.444	0.0003^
h62	00101010110		545	8	0	0.0075	0.726	0.355	0.0415

[1] DTE – dairy tick experiment, BRM – Brahman. Between parenthesis are the number of haplotypes reconstructed for each sample.
[2] N0 number of animals with zero copies of the haplotype, N1 number of animals with one copy of the haplotype, N2 number of animals with two copies of the haplotype.
[3] Proportion of residual variance explained by the common haplotype.
[4] Haplotype substitution effect in phenotypic standard deviations (tick count for the DTE and tick score for BRM).
[5] Standard error of α.
^ Significant after Bonferroni correction.
