# Supplementary material for: Haplotypes that include the integrin alpha 11 gene are associated with tick burden in cattle
Source: BMC Genet. 2010 Jun 21;11:55. doi: 10.1186/1471-2156-11-55 (PMC2905322; doi:10.1186/1471-2156-11-55)
Supplement: Additional file 4 — Description of the haplotypes reconstructed using 10 loci and the 3-locus sliding window. Description of the haplotypes reconstructed using 10 loci and the 3-locus sliding window. [file 1471-2156-11-55-S4.PDF]

Table S4. Description of the haplotypes reconstructed using 10 loci and the 3-locus sliding window.

SNP number[1]	Haplotype number	Code
1,2,3,4,5,6,7,8,9,11	h1	110100001 - 0
	h2	110101010 - 0
	h3	110110010 - 0
	h4	110110101 - 0
	h5	110111010 - 0
	h6	110111011 - 0
	h7	110111100 - 0
	h8	110111110 - 0
	h9	110111110 - 1
	h10	110111111 - 0
	h11	110111111 - 1
	h12	111111011 - 0
	h13	111000001 - 0
	h14	111000010 - 0
	h15	111000011 - 0
	h16	111000011 - 1
	h17	111000110 - 0
	h18	111000110 - 1
	h19	111000111 - 0
	h20	111000111 - 1
	h21	111010010 - 0
	h22	111010010 - 1
	h23	111010101 - 0
	h24	111010110 - 0
	h25	111010110 - 1
	h26	111011010 - 1
	h27	111011011 - 0
	h28	111011110 - 0
	h29	111011110 - 1
	h30	111011111 - 0
	h31	100111010 - 0
	h32	100111110 - 0
	h33	100111111 - 0
	h34	101011111 - 0
	h35	010111110 - 1
	h36	011111011 - 0
	h37	011111111 - 0
	h38	011000110 - 0
	h39	011000111 - 0
	h40	011000111 - 1
	h41	011010101 - 0
	h42	011010110 - 0
	h43	011011011 - 0
	h44	011011110 - 1

	h45	011011111 - 0
	h46	000100111 - 1
	h47	000101110 - 0
	h48	000110010 - 0
	h49	000110111 - 1
	h50	000111010 - 0
	h51	000111010 - 1
	h52	000111011 - 0
	h53	000111110 - 0
	h54	000111110 - 1
	h55	001000111 - 1
	h56	001010010 - 0
	h57	001010101 - 0
1,2,3	h1	110 - - - - - - - -
	h2	111 - - - - - - - -
	h3	100 - - - - - - - -
	h4	101 - - - - - - - -
	h5	010 - - - - - - - -
	h6	011 - - - - - - - -
	h7	000 - - - - - - - -
	h8	001 - - - - - - - -
2,3,4	h1	- 101 - - - - - - -
	h2	- 111 - - - - - - -
	h3	- 110 - - - - - - -
	h4	- 001 - - - - - - -
	h5	- 010 - - - - - - -
3,4,5	h1	- - 010 - - - - - -
	h2	- - 011 - - - - - -
	h3	- - 111 - - - - - -
	h4	- - 100 - - - - - -
	h5	- - 101 - - - - - -
4,5,6	h1	- - - 100 - - - - -
	h2	- - - 101 - - - - -
	h3	- - - 110 - - - - -
	h4	- - - 111 - - - - -
	h5	- - - 000 - - - - -
	h6	- - - 001 - - - - -
	h7	- - - 010 - - - - -
	h8	- - - 011 - - - - -
5,6,7	h1	- - - - 100 - - - -
	h2	- - - - 101 - - - -
	h3	- - - - 110 - - - -
	h4	- - - - 111 - - - -
	h5	- - - - 000 - - - -
	h6	- - - - 001 - - - -
	h7	- - - - 010 - - - -
6,7,8	h1	- - - - - 001 - - -
	h2	- - - - - 000 - - -

	h3	- - - - - 011 - - -
	h4	- - - - - 010 - - -
	h5	- - - - - 101 - - -
	h6	- - - - - 111 - - -
	h7	- - - - - 110 - - -
7,8,9	h1	- - - - - - 010 - -
	h2	- - - - - - 011 - -
	h3	- - - - - - 001 - -
	h4	- - - - - - 110 - -
	h5	- - - - - - 111 - -
	h6	- - - - - - 100 - -
	h7	- - - - - - 101 - -
8,9,10	h1	- - - - - - - 001 -
	h2	- - - - - - - 011 -
	h3	- - - - - - - 101 -
	h4	- - - - - - - 100 -
	h5	- - - - - - - 111 -
8,9,11	h1	- - - - - - - 10 - 0
	h2	- - - - - - - 10 - 1
	h3	- - - - - - - 11 - 0
	h4	- - - - - - - 11 - 1
	h5	- - - - - - - 00 - 0
	h6	- - - - - - - 01 - 0
9,10,11	h1	- - - - - - - - 010
	h2	- - - - - - - - 011
	h3	- - - - - - - - 000
	h4	- - - - - - - - 110
	h5	- - - - - - - - 111

[1] SNP ids: 1- rs29025980, 2- rs43616884, 3- rs29025985, 4- rs29025981, 5- rs41594962, 6-ss161109814, 7- 161109807, 8- 161109797, 9- rs29023635, 10- rs29023639, 11- rs29014770
